# Supplementary material for: Mobility as the Purpose of Postural Control
Source: Front Comput Neurosci. 2017 Jul 27;11:67. doi: 10.3389/fncom.2017.00067 (PMC5529402; doi:10.3389/fncom.2017.00067)
Supplement: Supplementary file 1 [file DataSheet1.pdf]

Appendix

1 Torque induced by muscular contraction

1.1 Torques of the external forces

When someone is standing on the ground, there are two external forces exerted on them: the person's weight and the ground reaction force.

Gravity exerts a downwards vertical force whose magnitude is the person's weight (their mass times the gravity of Earth). The point of application of the person's weight is called the centre of mass and noted CoM. If the CoM is vertically aligned with the ankles, then the person's weight does not exert a torque around the ankles (Fig. 1A). If it is forward of the ankles, then the weight exerts a forwards torque which is equal to the weight times the horizontal distance between the CoM and the ankles (Fig. 1B).

The ground supports the person's weight, therefore, as long as the CoM remains at the same height, the vertical component of the ground reaction force is of equal magnitude but of opposite direction to the person's weight. The ground reaction torque is therefore equal to the weight times the horizontal distance between the ankles and the point of application of the ground reaction force, called the centre of pressure and noted CoP.

The net torque around the ankles is thus determined by the horizontal distance between the CoP and the CoM: if they are vertically aligned, there is no net torque (Fig. 1 A and B), if the CoP is forwards of the CoM, then there is a net backwards torque (Fig. 1 C and D), and if the CoP is backwards of the CoM, then there is a net forwards torque around the ankles.

Such torque induces a change in the person's rotational momentum around their ankles, which is the sum over their body segments of the segment's mass, times its distance to the ankle, times its rotational speed (its speed perpendicularly to the axis joining it and the ankle).

1.2 Lower leg muscle contraction changes the ground reaction torque

We will show that only the forces exerted by the lower leg muscles onto the foot may change the ground reaction torque around the ankles.

In order to understand how the internal forces induced by muscular contraction may affect the ground reaction force, we shall decompose the body and consider only the foot (Fig. 2). If the foot is on a rigid support and does not slip, then it can neither translate, nor rotate around the ankle. Therefore, both the sum of forces and the sum of torques around the ankle must be zero. The forces exerted onto the foot are the ground reaction force (see the red arrow in Fig. 2), the foot's weight (which is negligible compared to the other forces), and the forces exerted by the lower leg onto the foot through on the one hand the muscles which attach onto the foot (see the blue arrows in Fig. 2), and on the other hand the bones, which exerts no torque around the ankles since it is applied at the ankles (see the green arrow in Fig. 2).

Thus, as long as the ground prevents the foot from moving, the torque of the ground reaction force around the ankle is exactly the opposite of that of the muscles of the lower leg. When the calf

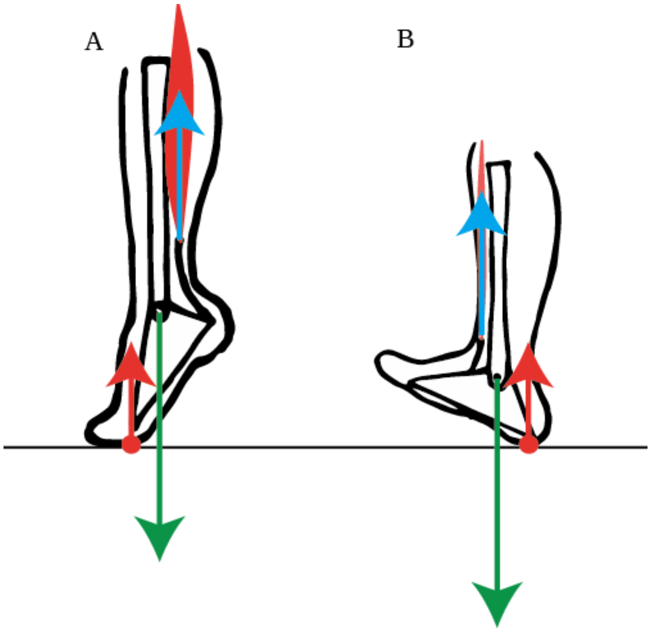

Fig. 10 Limited ground reaction torque

A. When the backwards torque exerted by the calf muscles exceeds the product of the person's weight and the distance between the ankles and toes, the person rises onto their toes. B. When the forwards torque exerted by the shin muscle exceeds the product of the person's weight and the distance between the ankle and heel, the person rocks onto their heels.

muscles contract, this pulls the heel upwards through the Achilles tendon (Fig. 2A). If the foot were in the air, it would rotate around the ankle joint bringing the toes down. Since the foot is against rigid ground, the ground resists the rotation of the foot by exerting backwards torque on the foot. Thus, any increase in the force that the calf muscles exert on the heel is instantly translated into an increase in the backwards torque of the ground reaction force on the entire body. As we have seen, as long as the CoM remains at the same height, the vertical component of the ground reaction force is of equal magnitude but of opposite direction to the person's weight. Since the magnitude of the vertical component of the ground force does not change, contraction of the calf muscles can only induce backwards ground reaction torque by shifting the CoP forwards (Fig. 2A). Likewise, any increase in the force of the shin muscle is instantly translated into an increase in the forwards torque of the ground reaction force on the entire body, through a backwards shift in the CoP (Fig. 2C).

1.3 The ground reaction torque is limited by the extent of the foot

However, the CoP cannot move further forwards than the toes. Thus if the contraction of the calf muscle exerts a torque that is larger than the product of the person's weight and the distance between their ankle and toes, then the foot can no longer remain immobile: the foot must then rotate around the toes. Indeed, when subjects are asked to rise onto their toes, they perform this movement with a burst of contraction of their calf muscles (Fig. 10A, Nardone and Schieppati, 1988). Likewise, shin muscle contraction induces forwards ground reaction torque by shifting the CoP backwards. However, the CoP cannot move further backwards than the heel. Thus when subjects are asked to rock onto their heels, they perform this movement with a burst of contraction of their shin muscle (Fig. 10B, Nardone and Schieppati, 1988).

The potential ground reaction torque is therefore limited by the extent of the foot: the forwards torque is limited to the product of the person's weight and the distance between the heels and the ankles, and

the backwards torque is limited to the product of the person's weight and the distance between the ankles and toes.

1.4 The net torque is limited by the position of the CoM

The ground reaction torque changes instantly when the torques exerted by the lower leg muscles on the foot change, but it is limited by the extent of the foot. The torque of weight on the other hand can only be changed by displacing the CoM forwards or backwards, which cannot be done instantly but first requires the sum of the external forces to accelerate the CoM horizontally. Therefore, at a given instant, the potential net torque that can be induced by muscular contraction is limited by the position of the CoM: the net forwards torque is limited to the product of the weight and the distance between the CoM and the heels, whereas the net backwards torque is limited to the product of the weight and the distance between the CoM and the toes.

2 Horizontal acceleration of the CoM

We will now consider the horizontal acceleration of the CoM. Since the person’s weight is vertical, only the ground reaction force may accelerate the CoM horizontally.

2.1 Acceleration of the CoM induced by mucsular contraction

The contraction of the dorsal muscles causes the trunk to rotate backwards around the hips (Fig. 11A). This backwards acceleration of the mass of the trunk implies that the trunk pushes forwards on the hips, which are therefore accelerated forwards. The dorsal trunk muscles do not exert torque on the foot around the ankles, therefore they do not induce a change in the ground reaction force. The person’s rotational momentum around their ankles is therefore unchanged. The increase in backwards rotational momentum around the ankles due to the backwards acceleration of the head must therefore be compensated by an equal increase in forwards rotational momentum due to the forwards acceleration of the hips. Since the head is further from the ankles than the hips are, and since rotational momentum is proportional to distance, this implies that the forwards acceleration of the hips exceeds the backwards acceleration of the head, such that the CoM is accelerated forwards (Fig. 11A).

The contraction of the calf muscles causes the legs to rotate backwards. However, the calf muscles do not exert torque on the trunk around the hips. Therefore, if only the calf muscles contract, then the rotational momentum of the trunk around the initial position of the hips is unchanged: due to its inertia, the trunk therefore rotates forwards in the external frame of reference as the legs rotate backwards. The person therefore flexes at the hips (Fig. 11B). Moreover, the contraction of the calf muscles induces backwards torque from the ground reaction force and therefore increases the person’s backwards rotational momentum around the ankles. The increase in backwards rotational momentum around the ankles due to the backwards acceleration of the hips must therefore exceed the forwards rotational momentum due to the forwards acceleration of the trunk. This implies that the CoM is accelerated backwards (Fig. 11B).

Thus, contracting the dorsal trunk muscles accelerates the CoM forwards (Fig. 11A) and contracting the calf muscles accelerates the CoM backwards (Fig. 11B). In order to accelerate the CoM backwards at the initiation of a movement requiring both calf and dorsal trunk muscle contraction, the calf muscle contraction should therefore precede the dorsal trunk muscle contraction.

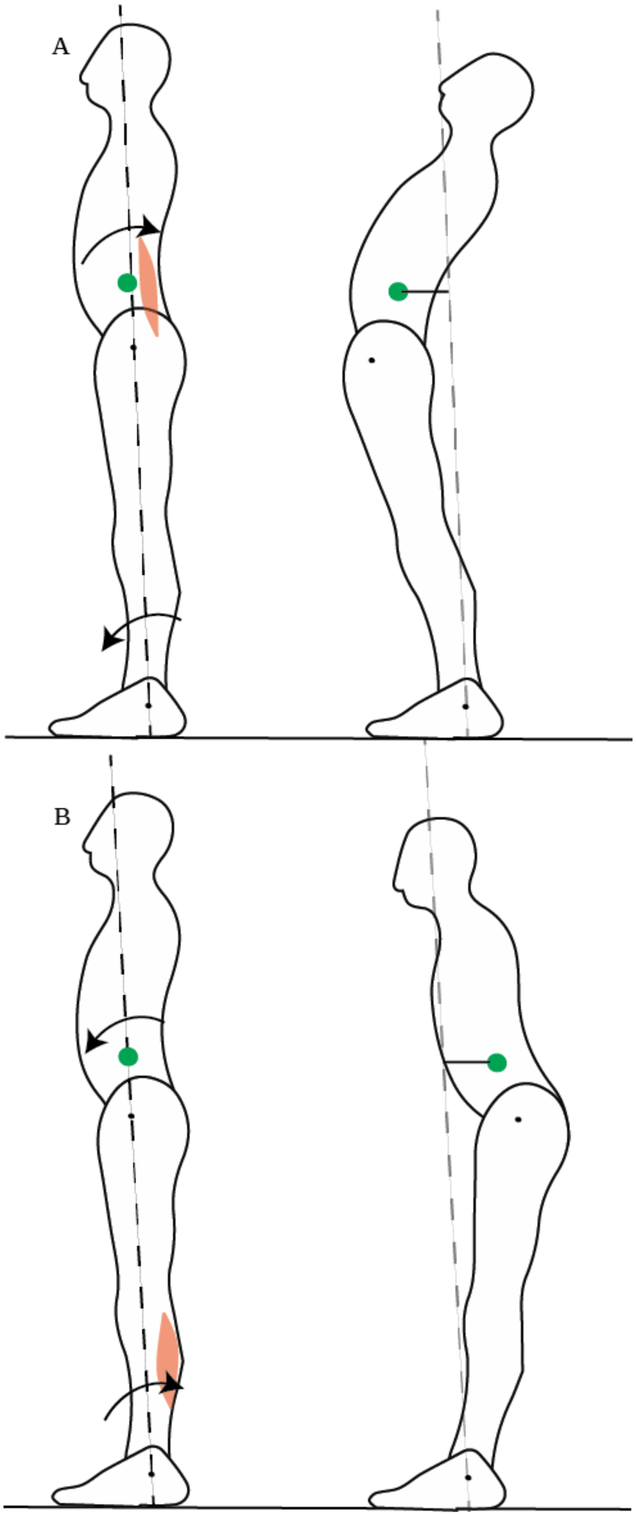

Fig. 11 CoM acceleration induced by muscular contraction

A. Dorsal trunk muscle contraction makes the trunk rotate backwards around the hips while the legs rotate forwards around the ankles, accelerating the CoM forwards. B. Calf muscle contraction makes the legs rotate backwards around the ankles while the trunk rotates forward around the hips, accelerating the CoM backwards.

2.2 Muscular contractions required for movement

Leaning the trunk forwards requires not only an increase in the trunk's forwards rotational momentum around the hips, through the contraction of the abdominal muscles, but also an increase in

the person’s total forwards rotational momentum around the ankles, through the contraction of the shin muscle. Thus, when leaning forwards, both the abdominals and the shin should be considered as “prime movers”, since they play the same role of providing torque for movement. If we take into account the knee joint, then the same analysis shows that leaning the trunk also requires the contraction of the thigh muscles. Moreover, the initial acceleration of the CoM requires a temporal sequencing of muscular contraction, with the lower leg muscles contracting first, then the thigh and finally the trunk muscles.

In order to straighten up after a platform translation, the person must rotate their entire body backwards around the ankles, keeping their legs and trunk aligned. This movement requires backwards rotational momentum of the body around the ankles, and therefore calf muscle contraction, but also backwards rotational momentum of the trunk around the hips, and therefore contraction of the dorsal trunk muscles. If we take into account the knee joint, then the same analysis shows that straightening up also requires contraction of the dorsal thigh muscles. Moreover, the initial acceleration of the CoM requires a temporal sequencing of muscular contraction, with the lower leg muscles contracting first, then the thigh and finally the trunk muscles.
